# Increased p16 and p53 protein expression predicts poor prognosis in mucosal melanoma

**DOI:** 10.18632/oncotarget.18367

**Published:** 2017-06-05

**Authors:** Hanbin Chen, Yangyang Li, Yin Long, Erjiang Tang, Rongrong Wang, Kate Huang, Congying Xie, Guorong Chen

**Affiliations:** ^1^ Department of Radiotherapy and Chemotherapy, The First Affiliated Hospital of Wenzhou Medical University, Wenzhou, Zhejiang, China; ^2^ Department of Pathology, The First Affiliated Hospital of Wenzhou Medical University, Wenzhou, Zhejiang, China; ^3^ Center for Translational Medicine, Yangpu Hospital, Tongji University School of Medicine, Yangpu, Shanghai, China

**Keywords:** p16 protein, p53 protein, mucosal melanoma, prognosis of mucosal melanoma

## Abstract

Primary mucosal melanoma (MM) is a rare, and aggressive, neoplasm with a poor prognosis. To date, few prognostic markers of MM have been well-defined. The aim of this study is to clarify the prognostic value of p53 and p16 proteins in predicting the clinical outcome of Chinese patients with MM.

A total of 59 MM samples were contained from biopsy specimens, and, expressions of p53 and p16 proteins were assessed by immunohistochemistry. Cox regression analysis was performed to investigate the association of these proteins with the overall survival of MM patients. Increased p16 expression was significantly associated with reduced survival at three years (P=0.039). Increased p53 expression correlates with reduced one-year (P=0.025), and, two-year survival (P=0.037).

Increased p53 and p16 protein expression may be helpful prognostic indicators for the management of these patients.

## INTRODUCTION

Mucosal Melanoma (MM) is a rare subtype of malignant melanoma representing approximately 1.3% of all melanomas [1–4]. Primary MM may arise from the head and neck (55%), female genital tract (18%), anorectal (24%), or urinary tract (3%), respectively [5]. Although the prevalence of this disease is relatively low, it has become increasingly clear that primary MM leads to worse outcome than other subtypes of melanoma. According to the data from a large, US registry study, the mean five-year overall survival rate for primary MM is only 25%; substantially lower than the 80% survival rate for cutaneous melanoma and 75% for ocular melanoma [5]. Stratified by anatomic sites, five-year overall survival rate for primary MM varies with 30% for the head and neck, 10% for the female lower genital tract and 20% for the anorectum [5]. This suggests that primary MM is a biologically-heterogeneous disease with wide variations in prognosis.

p53 is a tumor suppressor gene that plays a key role in maintaining genome stability and regulating cell cycle, apoptosis, differentiation and senescence [6]. It operates as a redox-sensitive transcription factor where ROS modifies p53 cysteine residues that affects transcriptional activity, and, stimulates a number of signaling pathways (APE-1/Ref1, ASK1/-p38, MAPK, PTPs-PKC8) [7]. p53 generally mutates in cancer leading to dysregulation of downstream genes [8, 9]. Many tumor-derived p53 mutants cause inactivation of p53 inducible protein 3 leading to accumulation of p53 and poor prognosis in a wide range of cancers [10, 11]. High levels of p53 also induce downregulation of superoxide dismutase that promotes apoptosis [12].

p16 is a kinase inhibitor encoded by the INK4a/CDKN2A gene. It binds to cyclin-dependent kinases (CDKs) CDK4 and CDK6, inhibits the phosphorylation of RB, resulting in cell cycle arrest, and, suppressing cell proliferation [13]. p16 overexpression occurs at the invasive margin in endometrial, colorectal and basal cell carcinoma suggesting a role in invasion [14–17]. It also plays a role in apoptosis and angiogenesis; restoring p16 ^Ink-4a^ results in down-regulation of vascular endothelial growth factor in different cell lines, and, inhibition of angiogenesis in malignant gliomas [18]. Interestingly, overexpression of p16 has been observed in a number of cancer cell lines in a p53 dependent [19, 20] or p53-independent manner [21, 22]. Taken together blocking cell proliferation, invasion and angiogenesis suggests a role for p16 ^Ink-4a^ as a “universal suppressor” in cancer cells.

Similarly, p16 is also significantly associated with cancer prognosis [23, 24]. Most malignant tumors over-expressing p16 ^Ink-4a^ do not demonstrate constant overexpression suggesting that they may bypass cell senescence in malignant disease. p16 overexpression in colon adenocarcinoma correlates with features of poor prognosis, and, in breast cancer, 20% of tumors overexpress p16 correlating with high grade and negative estrogen receptor status [22]. In that study p16 overexpression was also associated with p53 expression, vascular invasion and lack of progesterone receptors.

TNM staging is proposed by NCCN guidelines to predict prognosis and guide therapy for MM. Although this staging system takes into account the impact of tumor size and its metastasis to lymph node and other organs on prognosis, it is insufficient to guide individualized treatment due to a lack of prognostic markers. Therefore, it is important to identify factors capable of stratifying MM patients and predicting their outcomes. The purpose of this study is to assess the predictive value of p16 and p53 in Chinese MM patients.

## RESULTS

### Characteristics of MM patients

The clinical data of the 59 MM patients are summarized in Table 1. Overall, the median age of the patients is 73 years (range 31 to 96). There were 26 female and 33 male patients recruited to the study. The primary lesions were distributed in the head and neck (47/59, 79.7%), gastrointestinal system (8/59, 13.6%), genitourinary systems (3/59, 5.1%) lung (1/59, 1.7%), respectively. Head and neck MM patients consisted of 37 cases in the nasal cavity, 5 cases in the paranasal sinuses, 3 cases in the ocular orbit, and, 2 cases in the oral cavity.

**Table 1:** Characteristics of patients

Characteristics		Number of patients(%)	p53			p16		
			Low^#^	High^##^	P value*	Low^#^	High^##^	P value*
Total patients		59	47(79.7)	12(20.3)		28(47.5)	31(52.5)	
Age (year), median(range)		73(31-96)						
	≤73	30(50.8)	23(76.7)	7(23.3)		15(50.0)	15(50.0)	
	>73	29(49.2)	24(82.8)	5(17.2)	0.748	13(44.8)	16(55.2)	0.796
Gender, N (%)								
	Male	33(55.9)	28(84.8)	5(15.2)		16(48.5)	17(51.5)	
	Female	26(44.1)	19(73.1)	7(26.9)	0.336	12(46.2)	14(53.8)	1
Anatomic sites								
	Head and neck	47(79.7)	38(80.9)	9(19.1)		21(44.7)	26(55.3)	
	Gastrointestine	8(13.5)	5(62.5)	3(37.5)		5(62.5)	3(37.5)	
	Genitourinary	3(5.1)	3(100.0)	0		2(66.7)	1(33.3)	
	Lung	1(1.7)	1(100.0)	0	0.486	0	1(100.0)	0.542
Survival, N(%)								
	Alive	19(32.2)	17(89.5)	2(10.5)		10(52.6)	9(47.4)	
	Death	40(67.8)	30(75.0)	10(25.0)	0.303	18(45.0)	22(55.0)	0.781
One-year survival								
	Survival	40(70.2)	35(87.5)	5(12.5)	0.025*	23(57.5)	17(42.5)	0.081
Two-year survival								
	Survival	23(46.0)	20(87.0)	3(13.0)	0.037*	13(56.5)	10(43.5)	0.181
Three-year survival								
	Survival	13(28.3)	11(84.6)	2(15.4)	0.061	9(69.2)	4(30.6)	0.039*

^#^Low indicates the intensity score of p53 or p16 expression as no staining or weak staining.

^##^High indicates the intensity score of p53 or p16 expression as moderate or strong staining.

* P-value ≤0.05 was considered statistically significant.

### Protein expression of p16 and p53 in MM tissues

**Figure 1:**
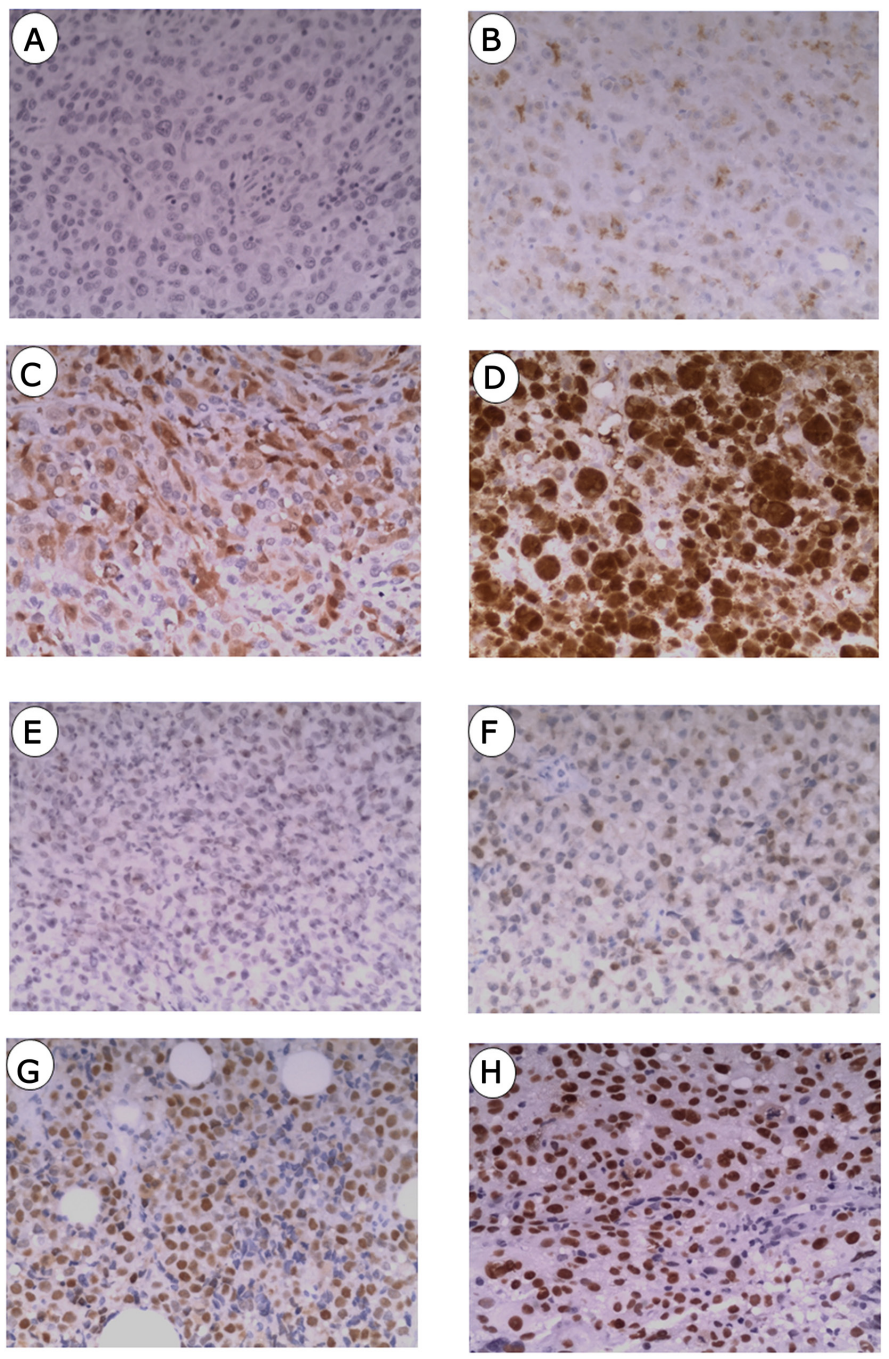
Immunostainings of p16 and p53 in mucosal melanoma **(A)** Negative staining of p16. **(B)** Weak staining of p16. **(C)** Moderate staining of p16. **(D)** Strong staining of p16. **(E)** Negative staining of p53. **(F)** Weak staining of p53. **(G)** Moderate staining of p53 protein. **(H)** Strong staining of p53.

Expression of p16 was detected in both the nucleus and cytoplasm, whereas p53 protein was confined to the nucleus (Figure 1). Patients with low p16 expression accounted for 47.5%, compared with 52.5% patients with high expression of p16 (Table 1). High expression of p53 was scored in 20.3% (12/59) of MM patients, which is much lower than that of patients with low expression of p53. Neither p16 nor p53 significantly correlated with the clinical characteristics.

### Association of p53 and p16 with survival

**Figure 2:**
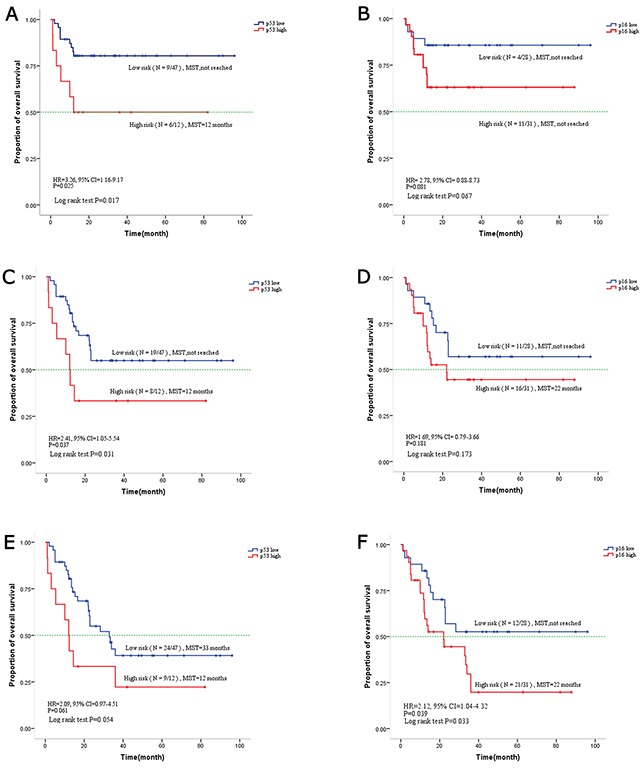
Kaplan-Meier curve for overall survival based on p16 and p53 expression in mucosal melanoma **(A)** One-year overall survival based on p53. **(C)** Two-year overall survival based on p53. **(E)** Three-year overall survival based on p53. **(B)** One-year overall survival based on p16. **(D)** Two-year overall survival based on p16. **(F)** Three-year overall survival based on p16.

Patients with high p53 expression had unfavorable one-year (HR, 3.26, 95%CI, 1.16-9.17, p=0.025), two-year (HR, 2.41, 95%CI, 1.05-5.54, p=0.037) and three-year (HR, 2.09, 95%CI, 0.97-4.51, p = 0.061) survival rates (Figure 2). Patients at high risk showed shorter mean survival times (12 months) in contrast to patients at low risk with longer mean survival times (33 months). Patients with a low expression of p16, had better three-year survival compared to patients with high p16 expression (HR, 2.12, 95%CI, 1.04-4.32, P=0.039) (Figure 2). The mean survival time of patients with low p16 expression was over 36 months compared to 22 months for patients with high p16 expression. Accordingly, patients with high p16 showed a trend toward poor one-year survival rates (HR, 2.78, 95%CI, 0.88-8.73, p=0.081) and two-year (HR, 1.69, 95%CI, 0.79-3.66, p=0.181).

## DISCUSSION

Increased expression of p53 and p16 were significantly associated with reduced overall survival in a cohort of 59 Chinese patients diagnosed with mucosal melanoma. For p16 this was limited to reduced overall, three year survival, and, for p53 it was reduced to one and two year survival (with a trend towards worse three year survival that did not reach statistical significance). These results are comparable with other reports of p16 and p53 overexpression in the literature though there are few reports discussing this particular tumor.

p53 expression appears abnormal in cutaneous and uveal melanomas [25–27]. Similar to cutaneous melanoma, high p53 expression was found in 20.3% (12/59) of MM samples in our study [28]. In general, over-expression of p53 in cancer is attributed to a series of p53 mutations which either result in dominant-negative effects over wild-type p53, or, acquire new activities contributing to tumor progression and drug resistance [29, 10, 30, 31]. Previously, Cox regression analysis showed that cytoplasmic p16 expression significantly correlated with poor survival in high-grade astrocytomas [32]. Similarly, another large retrospective study of 2197 breast cancer patients showed that strong p16 expression was significantly linked to shortened survival time [33]. Accordingly, high p16 expression was demonstrated to be significantly associated with a low three-year survival rate and showed a trend toward poor one-year and two-year survival rates (Figure 2).

It is important to bear in mind that this study used biopsy specimens to determine the expression of these proteins. There was not a study of specimens from a full-staging procedure so we cannot comment on the size, nature and extent of the initial tumor, in relation to the clinical outcomes. Also these patients only received adjuvant radiotherapy or chemotherapy if they developed metastatic disease so we cannot comment on the relationship between p16 and p53 and their importance in radiosensitive tumors.

A previous study in Korea reported an overall mean survival time of MM of 24.4 months, with two-year and five-year survival rates at 50.7% and 25.5% respectively [34]. We observed that positive p16 staining accumulated in both the nucleus and cytoplasm (Figure 1), consistent with other studies in MM and breast cancer [35, 36]. Intriguingly, p16 was detected in over 50% of samples in our study; relatively higher than the 25% detection rate in Western patients [37]. This suggests that the regulatory, molecular mechanisms leading to aberrant p16 expression may differ between races. Clearly we need to complete the follow-up of this group of patients to achieve a final conclusion about the utility of these markers in this rare tumor. There will be other groups of patients with different cancers that can be fully staged, where this combination of markers may provide useful predictive information about prognosis.

## PATIENTS AND METHODS

### Patients and tumor tissue samples

The current study included a total of 59 patients who were diagnosed as MM and underwent surgery between 2000 and 2015 in the First Affiliated Hospital of Wenzhou Medical University in Eastern China. Patient eligibility included a histopathologic diagnosis of MM, and, a complete set of clinical and follow-up information. Overall survival time was assessed beginning from the time of initial diagnosis to the last follow-up or, date of death. Pathologic observations were evaluated by two pathologists independently using the existing hematoxylin-eosin(H&E) slides from routine diagnostics. The study was reviewed and approved by the institutional review board.

### Histology and Immunohistochemistry

All surgical specimens from MM patients were studied by gross and microscopic examination. Gross appearances of MM samples included large polypoidal masses, with or without, melanin pigment. All tissue samples sent were fixed in 4% formaldehyde. Specimens were subject to subsequent steps of dehydration, paraffin embedding, sectioning, hematoxylin, eosin (HE) staining, and light microscopy. Histologic examination revealed intratumoral heterogeneity including epithelioid, spindled, and small cell cytomorphology. Melanoma was diagnosed directly when the tumor cells were melanin-rich, and, had immunohistochemical staining for HMB45, Melan A, S100, CK and Ki67 as previous described [38, 39]. Immunohistochemical staining was performed using the p53 primary antibody (Maxim, China) and p16 primary antibody (ZSGB-BIO, China) in the 59 samples according to the manufacturer’s instruction.

### Immunohistochemical scoring and analysis

The staining intensity was scored using the following scale: no staining (0), weak (1), moderate (2), and strong (3). The staining intensity scores were then evaluated by two pathologists. Immunoreactive score (IRS) was then used to determine the staining level by staining intensity, and this IRS was used to grade the protein expression. IRS over 1 was considered as high expression while IRS below or equal to 1 was considered as low expression.

### Statistical analysis

The statistical analysis was done using R program. The Kaplan-Meier method was utilized to graph the survival curves. The log-rank test was used to analyze the significant difference of Kaplan-Meier curves. The overall risk of death was estimated as hazard ratios (HRs) and a 95% confidence interval (CI) using the Cox Regression Model. The χ2 test was utilized to determine the difference of clinicopathologic features between pairs of groups. In all analyses, P-value ≤0.05 was considered statistically significant.
